# Treatment of Cholera by Hypodermic Injections of Warm Water

**Published:** 1866-11

**Authors:** Hermann Beigel


### Trsatment of Cholera by Hypodermic Injections of Warm Water.

BY HERMANN BEIGEL, M. D.

M. S., a widow of 44 years, had, since the outbreak of the present epidemic, been very much engaged in nursing cholera patients. On the 26th of August she did not feel quite well; and about one o'clock A. M., on the 27th, she was severely attacked by cholera, vomited very much, was purged severely, and subjected to violent cramps. In that condition she was received on the 27th into the cholera ward of the London Hospital. She was a well made woman, strong and muscular. The eyes lay deep in the orbits, and had a very distinct dark circle. The skin, particularly of the face and hands, was cold, inelastic, and wrinkled; the nails blue. The patient was extremely thirsty, frequently vomiting and purging, and up to twelve A. M., at which hour I first saw her, scarcely a few minutes free from cramps, so that she was constantly screaming with pain. The epigastric region was particularly painful. The case was, in the opinion of the house-surgeon, as well as of the matron, one of the worst which had been admitted into the hospital during the epidemic.

Temperature on admission, 36 3° cent.; pulse 112, very small, scarcely perceptible; respiration 22 in a minute. At two o'clock P. M. the temperature was 35.6 cent.; pulse 96; respiration 24; at six P. M. temperature 35.9° cent.; and at eleven P. M. temperature 36.0° cent., pulse 104, respiration 22. On the following day, at six A. M., the temperature was 53.8° cent., pulse not perceptible, respiration 28; at eleven A. M. temperature 35.5 cent., pulse not perceptible, respiration 30; at four P. M. temperature 34.9° cent., pulse not perceptible, respiration 36.

After examining the patient, I proceeded to inject warm water hypodermically. This operation was performed much more easily than I had imagined. In the calves, thighs, arms, and epigastric region seven ounces of water were injected, and so rapidly absorbed that I did not even find it necessary to put a piece of plaster to the little wounds caused by the syringe. Immediately after the completion of the injection the cramps ceased entirely, and did not return till the death of the patient. According to the report of the matron the thirst was also diminished, and the patient remained longer without asking for drink than she had done before the injections; the pulse became likewise more perceptible, and was reduced from 112 to 96. At half-past five o'clock P. M. I again injected four ounces and a half of warm water, and, according to the report, the vomiting and purging were not so frequent; but, as showu above, the principal sign — namely, the temperature — not only showed no improvement, but a decrease.

On the 28th, the general appearance of the patient was worse; vomiting and purging became, towards morning, again more frequent; and the skin was very cool, and void of elasticity. I injected five ounces of water, and the absorption still tóok place rapidly. In the course of the day the expression of the eyes became staring, the collapse increased, and the patient sank at about six P. M.

This case, although it ended fatally, affords some points of very great interest.

1.—It has not been known hitherto that such large quantities of fluid could safely be injected hypodermically.

2.—It is of great importance to know that, even in collapse, and when the stomach steadily refuses to retain its contents or anything that has been introduced into it, the skin is capable of absorbing fluids and bringing them into the current of circulation. Of that fact use may be made in other diseases, and nutrition carried on artificially till the alimentary tract is brought into a better condition.

3.—Experiments have been made to carry out the idea just mentioned by injecting broth and other fluids, as much as a pint at one spot. Such a method is physiologically not justifiable, and, therefore, will scarcely be crowned with success; on the contrary, it causes inflammation of the respective parts and gangrene of the skin, whilst injection of a considerable quantity of fluids at different spots, and of about twenty or thirty minims at a time, may be practiced without causing any considerable pain, and with the certainty of the separate small quantities of fluid immediately being absorbed and carried into the circulation.

4.—In the case described above the injection of seven ounces of water was effected in about an hour and a half; but, to perform that operation in a shorter time, I had an instrument manufactured by Messrs. Krohne & Sesemann, of Whitechapel Road, which I call an "injector," and by means of which several ounces and even a pint of fluid may be conveniently injected hypodermically, according to the principles I have stated, in a very short time.

5.—Should I have further opportunity of continuing this kind of treatment, the solution which I would choose for injection would be one containing ½ per cent. of phosphate of soda and ½ per cent. of common salt, that being the chemical composition of the rice-water evacuations as analyzed by Kletzinsky.

6.—Hypodermic injections, as I learn from several communications made to me after my short paper in the *Lancet* of 25th August, have been recommended also by different authors on the continent, and particularly by Dr. W. M. Gunning, of Amsterdam; and it really seems that this method, when properly applied, will at all events enable medical men to act more surely, and therefore more energetically, against the disease.—*Lancet*, Sept. 29, 1866.

